# Presynaptic Mitochondria Communicate With Release Sites for Spatio-Temporal Regulation of Exocytosis at the Motor Nerve Terminal

**DOI:** 10.3389/fnsyn.2022.858340

**Published:** 2022-05-12

**Authors:** Mario Lopez-Manzaneda, Andrea Fuentes-Moliz, Lucia Tabares

**Affiliations:** Department of Medical Physiology and Biophysics, School of Medicine, University of Seville, Seville, Spain

**Keywords:** mitochondria, synapse, exocytosis, calcium, synchronous release, asynchronous release, neuromuscular junction, active zones

## Abstract

Presynaptic Ca^2+^ regulation is critical for accurate neurotransmitter release, vesicle reloading of release sites, and plastic changes in response to electrical activity. One of the main players in the regulation of cytosolic Ca^2+^ in nerve terminals is mitochondria, which control the size and spread of the Ca^2+^ wave during sustained electrical activity. However, the role of mitochondria in Ca^2+^ signaling during high-frequency short bursts of action potentials (APs) is not well known. Here, we studied spatial and temporal relationships between mitochondrial Ca^2+^ (mCa^2+^) and exocytosis by live imaging and electrophysiology in adult motor nerve terminals of transgenic mice expressing synaptophysin-pHluorin (SypHy). Our results show that hot spots of exocytosis and mitochondria are organized in subsynaptic functional regions and that mitochondria start to uptake Ca^2+^ after a few APs. We also show that mitochondria contribute to the regulation of the mode of fusion (synchronous and asynchronous) and the kinetics of release and replenishment of the readily releasable pool (RRP) of vesicles. We propose that mitochondria modulate the timing and reliability of neurotransmission in motor nerve terminals during brief AP trains.

## Introduction

Synapses can broadly modulate their responses according to the pattern of the stimuli they receive, which has important consequences for information processing. Short-term synaptic plasticity is mostly presynaptic, and multiple mechanisms enhance or depress the synaptic output. Presynaptic Ca^2+^ is one of the main determinants of plasticity as it regulates exocytosis, endocytosis, and synaptic vesicle mobilization (Wu et al., 2014; Chamberland and Tóth, 2016; Leitz and Kavalali, 2016). For example, different electrical stimulation patterns generate distinct spatio-temporal increases in intracellular Ca^2+^ concentration [(Ca^2+^)_i_], which, in turn, determine the number and timing of vesicle fusions. Among the multiple modes of neurotransmitter release, spontaneous, phasic, and asynchronous, the last two are action potential (AP) dependent. Phasic (synchronous) release is triggered by the arrival of one or more AP to the nerve terminal and the subsequent rapid and transient influx of Ca^2+^ through voltage-gated calcium channels in active zones (AZ), which produces the fusion of primed synaptic vesicles with the plasma membrane on a submillisecond time scale. Following the fusion of vesicles, the AZs reorganize, and new vesicles are recruited and docked to the release sites so that neurotransmission can continue efficiently. Asynchronous release occurs during and after a stimulation train due to the accumulation of Ca^2+^ (residual Ca^2+^) at release sites (Atluri and Regehr, 1998).

The large capacity of mitochondria to sequester and release Ca^2+^ is essential in many cell types to maintain Ca^2+^ homeostasis for different functions, from muscle contraction to secretion (Pallafacchina et al., 2021). In nerve cells, mitochondria are highly sensitive to increases in cytosolic [Ca^2+^] (Chouhan et al., 2010; Ashrafi et al., 2019; Lopez-Manzaneda et al., 2021), making them good candidates to regulate synaptic activity and plasticity (MacAskill et al., 2010; Harris et al., 2012; Yang et al., 2021). Mitochondria have been shown to participate in the regulation of synchronous and asynchronous neurotransmitter release during an intense neuronal activity at motor nerve terminals (Tang and Zucker, 1997; David and Barrett, 2000, 2003; Talbot et al., 2003; Mironov and Symonchuk, 2006). However, the role of mitochondrial Ca^2+^ uptake in secretion properties during short-duration high-frequency AP bursts at mouse motor nerve terminals remained to be investigated.

Here, we used combined simultaneous real-time measurement of mitochondrial Ca^2+^ (mCa^2+^) and exo-endocytosis to analyze the spatial and temporal relationships between release sites and mCa^2+^ uptake during electrical nerve activity. Furthermore, intracellular synaptic potential recordings were used to examine the effects of inhibiting mCa^2+^ uptake by carbonyl cyanide m-chlorophenylhydrazone (CCCP) on synaptic transmission. We found that mitochondria participate in establishing synaptic properties even during short bursts of AP.

## Material and Methods

### Animal Model

We generated an FVB/NJ mouse line that expressed the Synaptophysin-pHluorin (SypHy) protein endogenously in neurons under the Thy1.2 promoter^1^. SypHy mice appeared normal in size, weight, and behavior, and the morphology and functionality of their motor nerve terminals were indistinguishable from SypHy negative mice. The recordings were made in adult mice (2–3 months old). All experiments were carried out according to the guidelines of the Directive of the European Council for Laboratory Animal Care and the Animal Care and Ethics Committee of the University of Seville.

### Acute Neuromuscular Preparation

Mice were killed with 100% CO_2_. The levator auris longus (LAL) muscle, a fast-twitch muscle located in the rear part of the neck (Ojeda et al., 2020), was dissected as previously described (Tejero et al., 2016). The neuromuscular preparations were superfused with a solution of the following composition (in mM): NaCl 135, KCl 4, CaCl_2_ 2, MgCl_2_ 1, NaHCO_3_ 15, NaH_2_PO_4_ 0.33, and glucose 10. The solution was continuously gassed with 95% O_2_ and 5% CO_2_.

### Mitochondrial Calcium Probe Loading

For mitochondrial calcium measurements, we used the membrane-permeable Rhod-2 AM probe (Thermo Fisher, R1245MP, Spain). The acetoxymethyl ester (AM) form is preferentially restricted to mitochondria because of its net positive charge. Rhod-2 is a single wavelength Ca^2+^ indicator with a maximum absorption/emission wavelength of ~557/581 nm and a K_D_ of ~570 nM. As described before (Lopez-Manzaneda et al., 2021), the probe was dissolved in dimethylsulfoxide (DMSO) and diluted to a final concentration of 5 μM in the solution that perfused the neuromuscular preparation. The preparation was incubated with the probe for 30 min at room temperature. After incubation, the preparation was washed with the physiological solution in the absence of the probe for 30 min at 28°C–32°C.

### Live Imaging and Analysis

The nerve was stimulated using a suction electrode. Action potentials were elicited by square wave pulses of 0.15 ms duration and 2–10 mV amplitude at variable frequencies (20–100 Hz) and train durations (1–20 s)using an isolated pulse stimulator (A-M Systems, mo. 2100, USA). Muscle contractions were prevented by adding 10 μM D-tubocurarine (Sigma-Aldrich, T2379, Spain) to the bath solution. Intervals between trains were always ≥10 min unless otherwise stated to allow complete recovery of terminal resting values. Experiments were conducted at 28–32°C using a temperature controller (TC-344B) connected to a thermistor (SF-28 SloFlo, Warner Instruments, USA). Exo-endocytosis (SypHy) and mCa^2+^ (Rhod-2) images were acquired and analyzed similarly. SypHy and Rhod-2 were excited with a 488 nm laser line. The different emission signals were captured separately using a 525/50 nm emission filter for the SypHy signal and a 617/73 nm emission filter for the Rhod-2 signal. Both fluorescence signals were monitored with a Yokogawa CSU-X1 spinning disk system (3i, Germany) mounted on an upright BX61WI microscope (Olympus, Spain) equipped with a water-immersion LUMPlanFI objective (x60, NA: 0.9). The images were captured using an EM-CCD camera C9100-13 (Hamamatsu, Spain) with an effective number of pixels of 512(H) × 512(V) and a pixel size of 16 × 16 μm. The images were acquired up to four frames per second with commercial software (SlideBook^TM^ 5.0, 3i, Germany), only in the best focus plane since the 3D simultaneous acquisition was not possible given the low fluorescent intensity of SypHy and the fast rise of the Rhod-2 signals.

**Figure 1:**
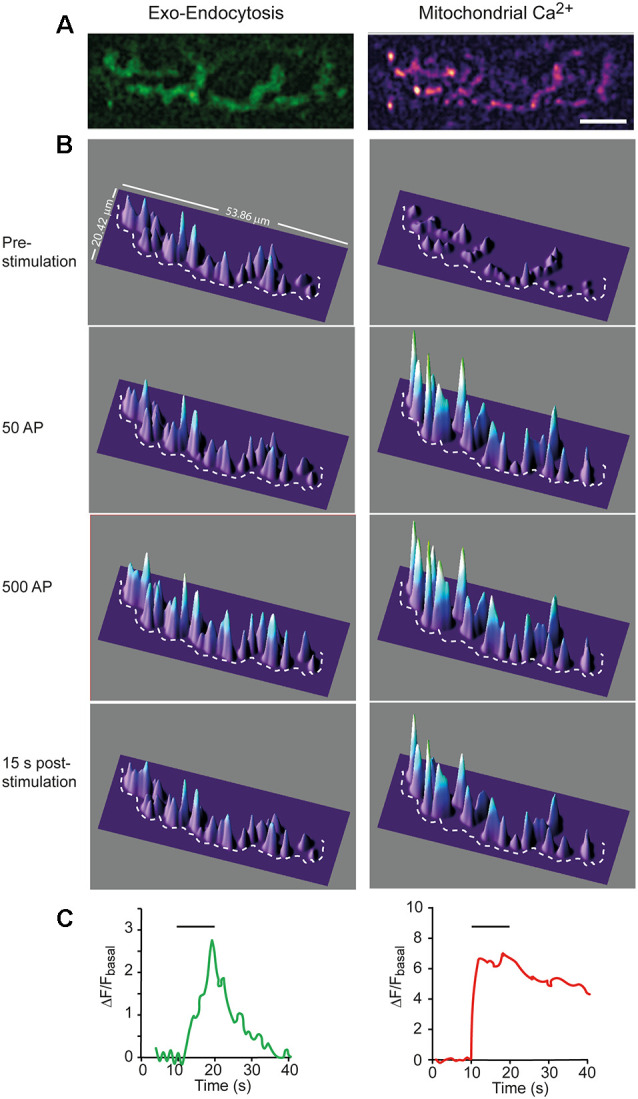
Simultaneous recording of hot spots of exocytosis and mCa^2+^ in a single nerve terminal. **(A)** Representative example of SypHy (left) and Rhod-2 (right) fluorescence signals recorded from a nerve terminal at the end of a 10 s stimulation train at 50 Hz. Calibration bar: 10 μm. **(B)** 3D intensity plots of the same terminal before, during stimulation (after 50 and 500 AP), and after stimulation. The dotted line partially displays the boundary of the terminal. **(C)** Mean intensity changes of SypHy (left graph) and Rhod-2 (right graph) vs. time at the terminal in **(A)**. Horizontal lines show the start and end of the stimulation train.

Before analysis, images were aligned using the automatic routine of the Slidebook program. Images were exported to FIJI (ImageJ) and split into two separate channels. The regions of interest (ROI) were outlined with a threshold-based macro routine, and the data were exported to Microsoft Office Excel. The fluorescence intensity of each ROI was corrected by subtracting the mean background level in the corresponding channel. Fluorescence intensity values were plotted vs. time to calculate different parameters. Correction for time-dependent loss of the SypHy signal, primarily due to photobleaching, was performed by subtracting the exponential fits of the resting-state fluorescence before and after recovery from the stimuli. Although each ROI was analyzed individually, the characteristics of the different ROI responses of the same nerve terminal were usually similar and thus averaged together for plotting the mean response (Figure 1C). Baseline fluorescence (F_basal_) was measured as the average fluorescence in ROIs before the stimulus (at least 10 frames). The change in fluorescence was expressed as ΔF (ΔF = F − F_basal_) or normalized to the baseline (ΔF/F_basal_). For mCa^2+^ (Rhod-2), the rise time was calculated as the time from 10 to 90% of the maximum fluorescence, and the decay time was calculated as the time required for the signal to return to half of its maximum value (t_1/2_). For the colocalization analysis between Rhod-2 and SypHy fluorescence signals, the JACoP (Bolte and Cordelières, 2006) plugin was used.

### Electrophysiology

The nerve was stimulated using a suction electrode. Action potentials were elicited by square wave pulses of 0.15 ms duration and 2–10 mV amplitude. A glass microelectrode (10–20 MΩ) filled with 3 M KCl was connected to an intracellular recording amplifier (TEC-05X; npi electronic, Germany) and used to impale single muscle fibers near the motor nerve endings. Evoked EPP and mEPP were recorded at room temperature (22°C–23°C), as previously described (Lopez-Manzaneda et al., 2021). Muscle contractions were prevented by including in the bath 2 μM μ-conotoxin GIIIB (Alomone Laboratories, C-270, Israel), a specific blocker of voltage-gated sodium channels in skeletal muscle. The recordings were sampled at 20 kHz, the mean amplitudes of the EPP and mEPP normalized to a resting membrane potential of −70 mV, and the EPP was corrected for nonlinear summation (McLachlan and Martin, 1981). Quantum content (*m*) was estimated by the direct method, which consists of recording mEPPs and EPPs (nerve stimulation 0.5 Hz) simultaneously and then calculating the ratio: *m* = Average EPP amplitude/Average mEPP amplitude. During a high-frequency train, *m* was estimated by calculating the ratio between each EPP and the average amplitude of the mEPPs for each experimental condition. To estimate the size of the RRP, *m* values during a train were plotted against time and fitted to a sequential model, as previously described (Ruiz et al., 2011). Briefly, the model assumes that quanta release on the stimulus came from the RRP, which subsequently was depleted along an exponential time course. The model also states that the recruitment process began after the first stimulus and rose sigmoidally to the plateau level as the original RRP became depleted. Then, we fitted the entire observed curve of quantal content along the train (*m* (t)) with two functions: a declining exponential plus a rising sigmoid, representing the contribution of the depletion of the RRP and the recruited vesicles, respectively: *m* (t) = A*exp(−t/B) + C/(1 + exp(−(t − D)/E)), where A represents initial *m*; B, time constant of RRP depletion; C, mean amplitude of the plateau; D, half-time of refilling; and E, steepness of the refilling time course. We constrained the sigmoid to start at zero. Integration of the first exponential gives the size of the RRP.

The mitochondrial membrane potential (ψ_m_) was depolarized with the protonophore carbonyl cyanide m-chlorophenylhydrazone (CCCP, 0.5–2 μM, Sigma-Aldrich C2759, Spain), which inhibits complex III of the electron transport chain preventing mCa^2+^ uptake and mitochondrial ATP synthesis. Mitochondrial complex V was inhibited with oligomycin (5 mg/ml, Sigma-Aldrich, Spain, O4876) to minimize ATP hydrolysis and partial ψ_m_ depolarization (David et al., 1998; David and Barrett, 2000). When possible, the same fiber was first recorded in the absence of drugs (control), then with oligomycin alone (after 20–30 min of incubation), and finally with oligomycin plus CCCP. The addition of oligomycin alone did not affect the amplitude or frequency of mEPPs compared to the control. The exposition time to CCCP was restricted to a maximum of 20–30 min to minimize the possible effects of decreasing/interrupting the oxidative ATP synthesis.

Asynchronous release during 50 Hz trains was estimated by counting the events between the second half of the inter-stimulus interval and multiplying by two since asynchronous events during the evoked responses could not be detected accurately (Talbot et al., 2003). However, in CCCP, when the frequency of mEPP became too high to be resolved, we calculated the area under the baseline elevation after correcting for nonlinear summation and divided by the pretrain averaged mEPP area, as previously described (Van der Kloot, 1990; David et al., 2003). Asynchronous release peak rates were calculated as the mean number of asynchronous events during the last 60 ms of 1-s trains and expressed per millisecond.

The probability of release for synchronous release upon the first shock of a stimulation train was calculated as the ratio of initial *m* vs. the estimated RRP size.

### Statistical Analysis

Statistical analysis of the imaging and electrophysiological data was performed using GraphPad Prism 5 (GraphPad Software). All values mentioned in the text and represented in the graphs are averages ± standard errors of the mean (SEM) unless otherwise stated. Parametric statistics were used whenever possible. The assumption of homogeneity of variances was assessed with the Levene test, using *α* = 0.05 as the cutoff. The Kruskal-Wallis rank-sum test was used when the distribution was not normal, followed by the *post hoc* Dunns multiple comparison test.

Given that the number of nerve terminals analyzed per condition was typically six, each terminal was treated as statistically independent. The results were considered statistically different when the P-value was <0.05. Data in parentheses (*n, N*): *n*, the number of nerve terminals (imaging experiments) or muscle fibers (electrophysiological experiments) per group; *N*, the number of mice per group. All reported experiments include the results of at least three animals per condition.

## Results

### Spatio-Temporal Relationship Between Exocytosis and mCa^2+^ Uptake

We simultaneously monitored mCa^2+^ with Rhod-2 AM and exo-endocytosis in adult transgenic mice expressing synaptophysin-pHluorin (SypHy; Lopez-Manzaneda et al., 2021). The acetoxymethyl (AM) ester group of Rhod-2, which facilitates its uptake, is removed by intracellular esterases, resulting in the selective accumulation of the calcium dye within mitochondria (David et al., 1998; Talbot et al., 2003).

Figures 1A,B show 2D and 3D images, respectively, for Syphy and mCa^2+^ signals in a representative nerve terminal, stimulated with a 500 AP train (50 Hz). The surface intensity plots of the SypHy signal (Figure 1B, green channel, left column) displayed multiple peaks distributed along the terminal surface, which amplitudes increased upon stimulation, especially in certain regions, which represent hot spots of exocytosis (Wu and Betz, 1999; Tabares et al., 2007; Gaffield et al., 2009; Cano et al., 2012; Lopez-Manzaneda et al., 2021), and remain stable with repeated trains (Tabares et al., 2007).

The time course of the SypHy increase was relatively slow. Figure 1B, second and third panels, shows the fluorescence increase, representing the excess of exocytosis over endocytosis, at 1 s (50 AP) and 10 s (500 AP) of the stimulation. When stimulation ceased, fluorescence decreased to the base level (lower panel) due to the endocytosis-reacidification process (Sankaranarayanan et al., 2000). Figure 1C (left graph) shows the average change in SypHy fluorescence vs. time at this terminal. The amplitude of the signal and the time constant of endocytosis (~7 s) were similar to what has previously been described in this synapse (Cano et al., 2012; Cano and Tabares, 2016).

Similarly, the mCa^2+^ signal was distributed along the terminal surface, although its rising kinetics was much faster (Figure 1B, right column and Figure 1C, right graph) than the SypHy signal. The mCa^2+^ signal stayed high (plateau) during the stimulation train (second and third panels). The plateau represents the dynamic equilibrium between Ca^2+^ uptake, reversible formation of Ca^2+^-phosphate complexes, and Ca^2+^ efflux (Gunter and Sheu, 2009). After stimulation, the signal slowly returned to the basal level, indicating mCa^2+^ release to the cytosol, a process mainly driven by the mitochondrial Na^+^/Ca^2+^ exchanger. The average rise time was 1.07 ± 0.21 s, and the decay half-time was 17.67 ± 2.38 s (*n* = 6), which is consistent with previous measurements from presynaptic mitochondria of postnatal mouse motor nerve terminals (Lopez-Manzaneda et al., 2021). The slow release of Ca^2+^ from mitochondria has been shown to transitorily elevate cytosolic Ca^2+^ and promote post-tetanic potentiation at the mouse and crayfish motor nerve terminals (Tang and Zucker, 1997; García-Chacón et al., 2006) and at hippocampal mossy fiber synapses (Lee et al., 2007). In our recordings, Ca^2+^ efflux from mitochondria was slower than the endocytosis time course. Then, it would be of interest to determine in future studies whether mCa^2+^ release participates in the regulation of endocytosis.

### Estimation of Inter-distances Between Mitochondria and Exocytosis Hot Spots by Quantitative Spatial Analysis

**Figure 2:**
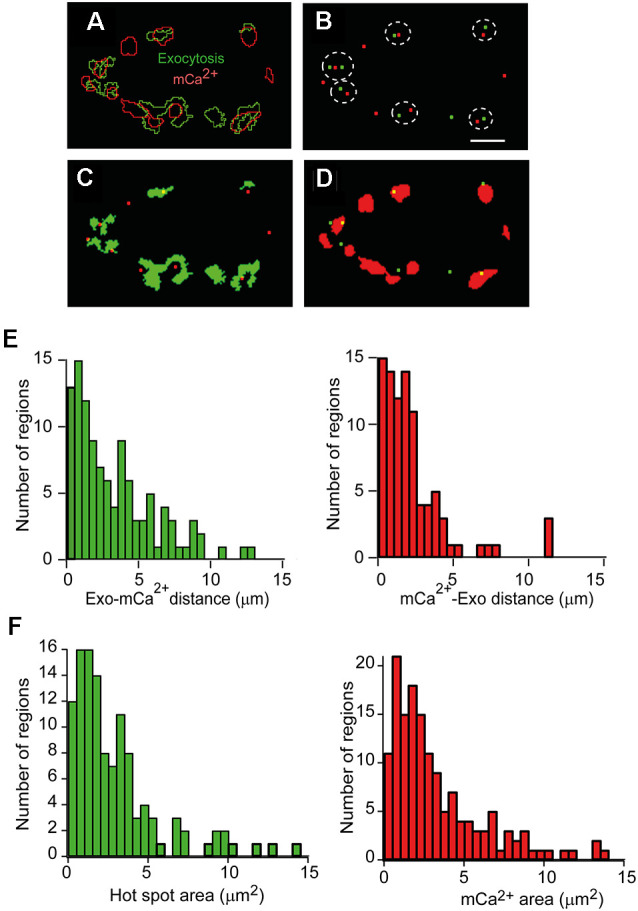
Quantitativeanalysis of the spatial relationship between exocytosis hot spots and mCa^2+^. **(A)** Representative image showing the outlines of hot spots of exocytosis regions (green) and mCa^2+^ increase regions (red) superimposed after a 500 AP train (50 Hz) of stimulation. **(B)** Spatial distribution of exocytosis hot spots centroids (green) and mCa^2+^ (red) centroids of the terminal in **A**. The dotted lines indicate the nearest neighbors between exocytosis hot spots and mCa^2+^. **(C)** Location of mCa^2+^ centroids (red) and the exocytosis hot spot areas (green). **(D)** Location of exocytosis hot spots centroids (green) and the mCa^2+^ areas (red). Calibration bar **(A–D)**: 5 μm. **(E)** Distance distribution between nearest-neighbor centroids in 11 terminals (four mice). Left: exocytosis-mCa^2+^ distance. Right: mCa^2+^-exocytosis hot spots distance. **(F)** Frequency histograms of the exocytosis hot spots areas (left) and mCa^2+^ region areas (right; 14 terminals, six mice) in each case.

We performed an object-based approach analysis (Bolte and Cordelières, 2006) to examine the spatial relationship between mitochondria and exocytosis hot spots. The procedure was as follows: First, the maximum increase in fluorescence (ΔF_exo_ and ΔFmCa^2+^) at both channels was obtained by subtracting the intensity of their respective last images during the stimulation from the average intensity obtained before stimulation (mean of 10 images). Then, the regions of interest (ROIs) were established using an intensity auto threshold macro (Lopez-Manzaneda et al., 2021), and the outlines were represented. Finally, the geometric center (centroid) of each ROI was obtained, and the inter-distances between objects were calculated (see “*Materials Methods*” Section). For example, Figure 2A shows the outlines from a representative nerve terminal containing 10 active mCa^2+^ regions (red outlines) and eight exocytosis hot spots (green outlines) merged in the same image.

Figure 2B shows the overlay of the centroids of the exocytosis hot spots (green) and the mCa^2+^ centroids (red) of the nerve terminal shown in Figure 2A. The dotted lines highlight the nearest neighbors’ centroids between channels. The centroids in one channel and the ROIs in the other channel, and* vice versa*, are also shown for this terminal (Figures 2C,D). In general, most exocytosis hot spots were close to one or two active mitochondrial regions.

The distance distributions of the closest neighbor centroids between channels in a total of 11 nerve terminals from four experiments are shown in Figure 2E. The distances are represented in two histograms since the number of centroids in one channel and the other was not the same for a given terminal. The most frequent inter-distances between centroids of different channels were ~0.5–1 μm, and ~61% of exocytosis hot spots centroids were within 2 μm of a mCa^2+^ centroid, and ~47% of mCa^2+^ centroids were within 2 μm from an exocytosis centroid.

Next, we wondered how many AZs could be within our exocytosis hot spots. In mouse motor nerve terminals, AZs are distributed throughout the surface terminal, and the mean density of AZs is ~2.4/μm^2^ (Fukunaga et al., 1983; Nishimune et al., 2004; Ruiz et al., 2011). Since, in our experiments, 80% of the hot spots had a surface area of ≤4 μm^2^ (Figure 2F, left histogram, 14 terminals, six mice), we estimate they may contain between ~1 and 12 AZs. For comparison, the size distribution of the mCa^2+^ regions measured in our experiments is shown in the right histogram of Figure 2F.

Together, these observations suggest that hot spots of exocytosis and a subset of mitochondria are located in sub-synaptic functional domains (tandems). This distribution may contribute to the local regulation of [Ca^2+^] and facilitate the delivery of ATP at places of high exocytosis activity.

### Presynaptic Mitochondria Efficiently Uptake Ca^2+^ in the Physiological Range of Neural Activity

**Figure 3:**
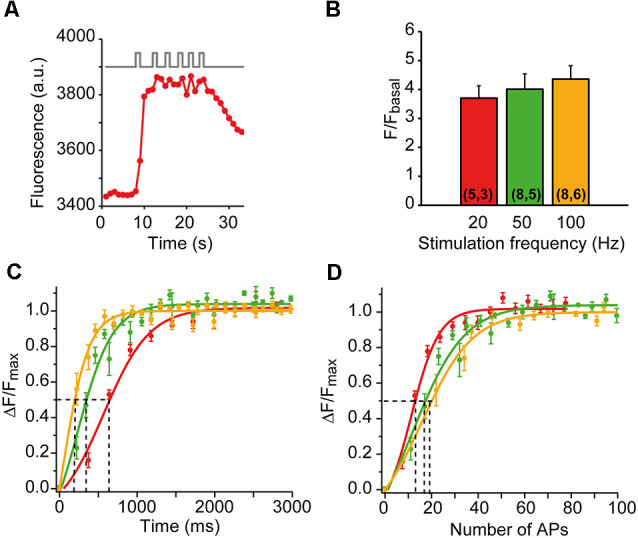
mCa^2+^responses to different patterns of nerve stimulation. **(A)** mCa^2+^ increases to six successive trains of 1-s duration at 50 Hz in a representative nerve terminal. **(B)** The average increase in fluorescence (ΔF/F_basal_) at 20, 50, and 100 Hz. The numbers of terminals and mice recorded are in parentheses. **(C,D)** Average fluorescence increase (ΔF/F_max_) as a function of time **(C)** and number of AP **(D)** at 20 (red symbols), 50 (green symbols), and 100 (yellow symbols) Hz. The solid lines are sigmoidal fit on the data. The time required to reach half-maximum mCa^2+^ (dotted lines in **C**) varies between 650 ms (20 Hz) and 190 ms (100 Hz). Half-increase in fluorescence is achieved with 13–19 AP (dotted lines in **D**). The apparent higher sensitivity of the response at 20 Hz in **(D)** is probably not such but reflects the time-dependent component of the response, as shown in **(C)**.

To examine the capacity of presynaptic mitochondria to respond to physiological stimuli, we used short-duration stimulation trains and different stimulation frequencies. For example, Figure 3A shows a representative nerve terminal stimulated with six consecutive 1-s trains at 50 Hz, spaced 2–3 s. The plateau amplitude was essentially reached with the first train, confirming the high sensitivity of mitochondria to a brief high-frequency burst of APs. The plateau was not due to probe saturation since permeabilization of the membrane with digitonin increased the fluorescence above the plateau amplitude (data not shown, and Lopez-Manzaneda et al., 2021).

We also compared the amplitude and the rising kinetics of the mCa^2+^ responses at different stimulation frequencies while maintaining the number of action potentials constant (500 AP). The mean amplitude of the responses increased little with the stimulation frequency (3.7-fold ± 0.43 at 20 Hz, 4.01-fold ± 0.53 at 50 Hz, and 4.36-fold ± 0.46 at 100 Hz; *n =* 5–8 nerve terminals at each frequency; Figure 3B), indicating a powerful calcium buffering system in the matrix (David, 1999; David et al., 2003). However, the rising kinetics of the signal was very sensitive to the stimulation frequency. Figure 3C shows the average time course of mCa^2+^ rise at each frequency, normalized to the plateau amplitude (ΔF/F_max_). The half-maximum amplitudes were reached at ~190 ms (100 Hz), ~360 ms (50 Hz), and ~650 ms (20 Hz), which correspond to the firing of 13–19 APs (Figure 3D). These results indicate that presynaptic mitochondria efficiently uptake calcium in the physiological range of neural activity of this synapse.

### Reduction of mCa^2+^ Uptake May Alter Exocytosis

To explore the role of mitochondria in modulating synaptic strength, we recorded exocytosis and mCa^2+^ before and after adding CCCP to the bath solution. CCCP inhibits complex III of the electron transport chain, which depolarizes the mitochondrial membrane potential (ψm), reduces stimulation-induced mitochondrial Ca^2+^ uptake, and increases cytosolic [Ca^2+^] (Tang and Zucker, 1997; David et al., 1998; David, 1999; Suzuki et al., 2002; David and Barrett, 2003; Talbot et al., 2003; Lopez-Manzaneda et al., 2021). In addition, ψm depolarization reduces the electromotive force used for mitochondrial ATP synthesis and produces additional ATP depletion associated with ATP synthase (complex V) reversion. To prevent this extra hydrolysis of ATP, we added oligomycin (5 μg/ml) before CCCP, an inhibitor of complex V that does not depolarize ψm (David et al., 1998).

**Figure 4:**
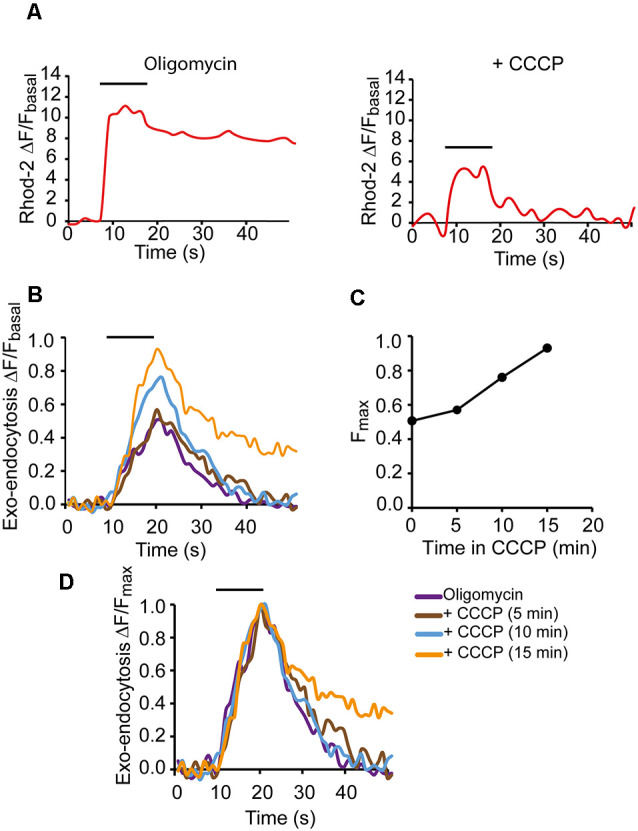
Modification of the secretory response in depolarized mitochondria.**(A)** mCa^2+^ responses to 500 AP stimulation (50 Hz) in oligomycin only solution (left graph) and with oligomycin plus CCCP (right graph). **(B)** SypHy fluorescence increases in oligomycin alone (purple trace) and after 10 and 15 min in oligomycin plus CCCP (blue and yellow traces, respectively). Fluorescence increments were represented relative to the basal fluorescence in each case. **(C)** Fluorescent peak amplitudes of the SypHy signals vs. time in CCCP. **(D)** SypHy fluorescence for recordings in **(B)** normalized to their respective peak values to compare the kinetics of the responses. Horizontal lines show the start and end of the stimulation train (500 AP, 50 Hz).

Figure 4 shows a representative example of a nerve terminal where mCa^2+^ and exocytosis were recorded first in oligomycin alone and then in oligomycin plus CCCP. As expected, the mCa^2+^ response to a 500 AP stimulation train (50 Hz) decreased considerably in the presence of CCCP (Figure 4A). On the contrary, the exo-endocytosis response increased (transitorily) over time (Figure 4C), compared to the oligomycin response alone (Figure 4B, purple trace: after 23 min in oligomycin; brown, blue, and orange traces: 5, 10, and 15 min in oligomycin plus CCCP). Normalizing the responses to their peak amplitudes (Figure 4D) revealed similar rise and decay kinetics in oligomycin (purple trace) and up to 10 min in CCCP (blue trace), and only slower relaxation after 15 min of CCCP (orange trace). The increases in the amplitude of the responses suggested an increase in exocytosis due to the elevation of cytosolic Ca^2+^. However, this effect was followed by a marked decrease in the signal soon after, an effect varying greatly between different terminals. For this reason, we decided to further investigate the effect of mitochondria on secretion using an electrophysiological approach, which would allow us to use shorter stimulation trains and, therefore, minimize ATP consumption.

### Mitochondria Limit Asynchronous Release During Short-Duration AP Trains

**Figure 5:**
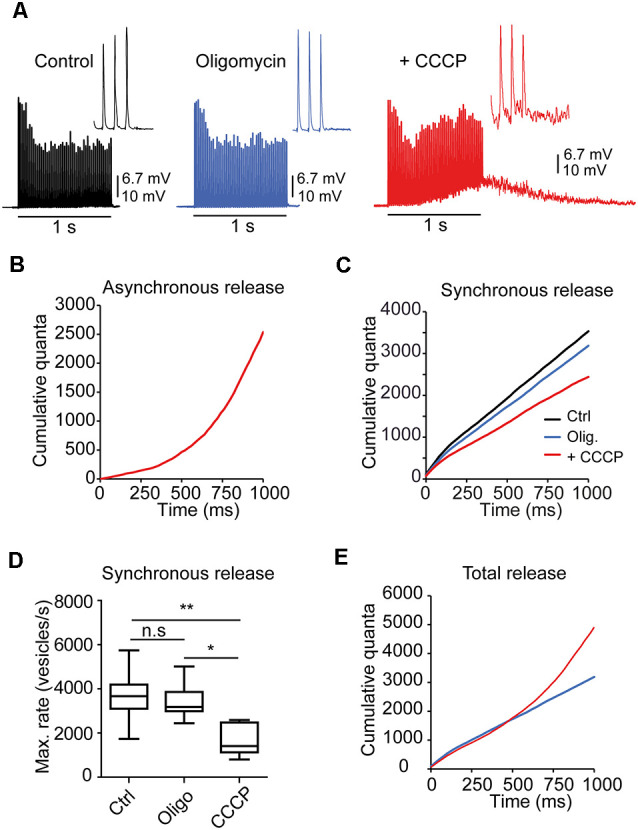
Mitochondria regulate asynchronous release during brief AP trains. **(A)** Representative responses to 1-s stimulation trains (50 Hz) in a fiber recorded in control solution, oligomycin, and oligomycin plus CCCP. Note the elevation on the baseline during and after the train due to the prominent increase in asynchronous release in the presence of CCCP. The insets display the last three EPPs of the trains for each condition. **(B)** Cumulative asynchronous release during synaptic activity in CCCP (the same fiber as in **A**, estimated by integration, as described in “*Materials and Methods*” Section. **(C)** Cumulative synchronous quanta release from the fiber in **(A)** in all three solutions. **(D)** Average cumulative synchronous quanta release in control, oligomycin alone, and oligomycin plus CCCP (24 fibers, eight mice) (P_Kruskal-Wallis_ = 0.0034). The box-whisker plot represents median (line), 25th–75th percentile (box), and min–max (whisker) for each condition. *:<0.05; **:<0.005; n.s., not significative. **(E)** Total quanta release (synchronous + asynchronous) in CCCP (red line). For comparison, release in oligomycin alone (blue line) is also shown.

To further elucidate the role of mitochondria in neurotransmission during short-duration high-frequency nerve activity, we recorded endplate potentials (EPP) and miniature endplate potentials (mEPP) in the control solution, then in oligomycin, and finally with oligomycin plus CCCP in the same fiber. Stimulation in the control solution produced the typical response consisting of a progressive decrease in the EPP amplitude from an initial value to a plateau that was approximately one-half of the initial amplitude (Figure 5A; left recording). Stimulation after 20 min of incubation with oligomycin (5 mg/ml) did not significantly change the response (Figure 5A; central recording) nor the amplitude of EPP and mEPP. However, 5 min after adding oligomycin + CCCP (1 μM), there was an elevation on the baseline (Figure 5A; right recording) during the second half of the stimulation train and several seconds after the train due to the buildup of asynchronous release (David and Barrett, 2003). This response was never observed in control or oligomycin alone.

Before each train, the mEPP rates were similar in control (2.55 vesicles/s) and oligomycin (2.67 vesicles/s) but slightly elevated in CCCP (6.87 vesicles/s), which may represent a small increase in resting [Ca^2+^]_i_.

Estimation of the amount of intra-train asynchronous events (see “*Materials Methods*” Section) in this fiber showed two orders of magnitude higher peak vesicle release rates in CCCP (~2,500 s^−1^, Figure 5B) than in control and oligomycin (~30 s^−1^), indicating that the mean survival time of a vesicle before fusing asynchronously changed from ~33 ms to a fraction of a ms after CCCP. On the contrary, on average, no statistical differences were found between control and oligomycin alone, which agrees with a previous report showing that the increase in asynchronous release with CCCP, in experiments lasting <1 h, is due to the increase in the [Ca^2+^]_i_ rather than inhibition of mitochondrial ATP synthesis (David and Barrett, 2003).

In contrast, cumulative synchronous release was reduced in CCCP compared to the control (Figure 5C). On average, the maximum vesicle release rate (Figure 5D) for synchronous release was approximately half in CCCP (1,649 ± 290 vesicles/s, *n =* 6 fibers) than in control (3,658 ± 346 vesicles/s, *n =* 10 fibers) or oligomycin alone (3,447 ± 277 vesicles/s, *n =* 8 fibers; P_Kruskal-Wallis_ = 0.0034; Figure 5D).

Typically, total cumulative release (synchronous + asynchronous) in CCCP (Figure 5E, red line) was equivalent to release in oligomycin alone (blue line) during the first 500 ms of stimulation, suggesting that a proportion of the RRP vesicles fused asynchronously (see “*Discussion*” Section). However, during the second part of the train, total release in CCCP exceeded by <1,725 vesicles evoked release in oligomycin that could obey fusions of secondary docked vesicles (Nagwaney et al., 2009) or to the acceleration of the recruitment rate (Lu and Trussell, 2000).

Together, these data suggest that fast mitochondrial Ca^2+^ uptake restricts Ca^2+^ building up within microdomains and maintains the equilibrium between synchronous and asynchronous release during short bursts of AP.

### Mitochondria Modulate Short-Term Plasticity and the Depletion and Refilling Kinetics of the RRP

Subsequently, we investigated whether mitochondrial uptake of cytosolic Ca^2+^ regulates short-term plasticity, vesicle loss and replenishment kinetics, and RRP size during short-duration AP trains. Since the degree of ψm depolarization produced by CCCP increases during the experiment (David et al., 2003; Talbot et al., 2003; Lopez-Manzaneda et al., 2021), we analyzed the modification of the release properties over time in CCCP in single fibers. For the analysis, we used a simple sequential kinetic model (Ruiz et al., 2011) that assumes that the primary docked vesicles (the RRP) would be the first to be released with repetitive nerve stimulation, that the RRP pool is depleted exponentially, and that the recruitment of new vesicles begins, increases, and maintains the plateau (Elmqvist and Quastel, 1965).

**Figure 6:**
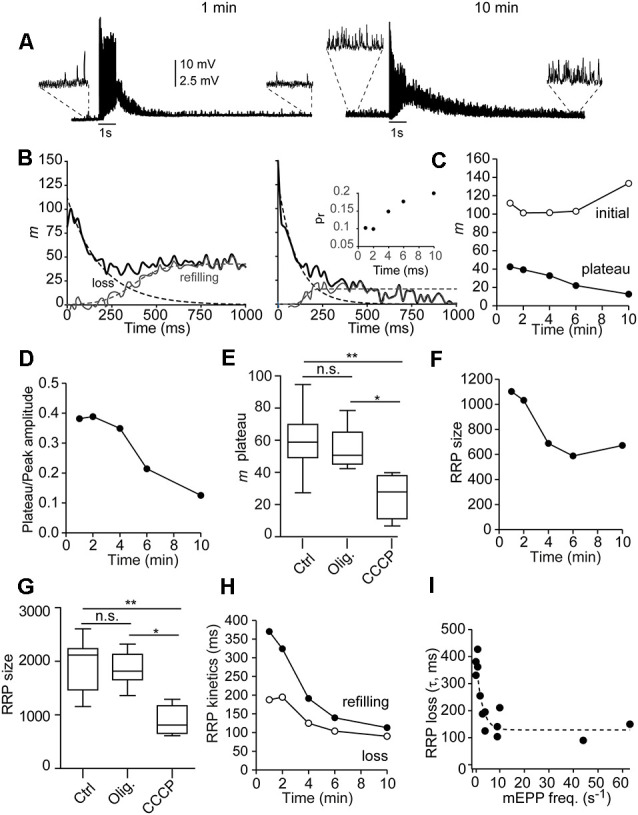
Mitochondriamodulate the depletion and refilling kinetics of the RRP andshort-term plasticity during physiological stimuli. **(A)** Representative EPP responses of a single fiber to two 1-s stimulation trains, 1 and 10 min after adding CCCP to the recording chamber. Note the high frequency of asynchronous release before (insets), during, and after (insets) each train. **(B)** Curve fitting of *m* values during the trains (thick lines) to a function of the sum of exponential decay of the RRP plus the sigmoidal rise of recruitment (dotted lines) of the recordings in 6A for estimation of the RRP size, RRP depletion, and recruitment time. The inset on the right graph displays the probability of release (*p_r_*) vs. time in the CCCP, estimated by dividing the number of quanta released with the first by the size of the RRP for each train. **(C)** Quanta released at the initial of the train (open symbols) and plateau amplitude (filled symbols) after 1, 2, 4, 6, and 10 min in CCCP. **(D)** Relationship between the plateau and the initial release amplitudes with time in CCCP. **(E,G)** Average plateau amplitude **(E)** and RRP size **(G)** in control (10 fibers, eight mice), oligomycin alone (eight fibers, six mice), and CCCP (5–10 min, six fibers, four mice). The box-whisker plots represent median (line), 25th–75th percentile (box), and min–max (whisker) for each condition (P_Kruskal-Wallis_ = 0.0018). *: <0.05; **: <0.005; n.s.: not significative. **(F)** Decrease of the RRP over time in CCCP (same fiber as in **A**). **(H)** Acceleration of RRP depletion (τ_RRP_, open symbols) and vesicle recruitment (*t_1/2_*, filled symbols) over time in CCCP. **(I)** Correlation between the depletion of the RRP and the frequency of mEPP before the train. The data were fitted with an exponential function (dotted line).

Examples of representative responses to two 1-s stimulation trains in a representative fiber, 1 and 10 min after adding CCCP (2 μM), are shown in Figure 6A. Note the high rates of mEPPs in the recordings (insets). The analysis performed is illustrated in Figure 6B (Ruiz et al., 2011). The thicker lines represent the quanta released synchronously (*m*) for each EPP. The loss of RRP (dotted exponential lines in Figure 6B) was estimated by adjusting the values of *m* during the first nine-10 stimuli in the train to a decaying exponential (see “*Materials Methods*” Section). The recruitment time course was then estimated by subtracting the exponential decay of the RRP from the entire observed curve and fitting the resulting curve with a rising sigmoid (dotted lines).

Nerve terminals recorded in control or oligomycin alone usually presented facilitation (increase in EPP size with the first two-three consecutive stimuli) in response to 50 Hz trains. Facilitation was still present at the beginning of the incubation with CCCP (Figure 6B, left recording) but generally disappeared later (Figure 6B, right graph). Figure 6C (open symbols) shows the number of quanta released after the first shock (initial *m*) vs. time for this experiment. The inset in Figure 6B shows the change in the probability of release (*p_r_*), calculated as the ratio of initial *m vs.* the size of the pool. The increase in *p_r_* (from 0.1 to 0.2), is correlated with a ~40-fold increase in the pre-train mEPP rate in this fiber (from 1.2 to 47 vesicles/s), presumably caused by an elevation in residual [Ca^2+^] within microdomains (Jackman and Regehr, 2017).

In contrast, the plateau amplitude, which reflects the equilibrium between depletion and vesicle replenishment, progressively depressed over time in CCCP (Figures 6B,C, filled symbols). For example, in this fiber, the steady-state to initial quanta ratio changed from ~0.4 to ~0.12 over time (Figure 6D). On average, the plateau amplitude after 5–10 min in CCCP was approximately half (25.5 ± 5.4 quanta) than in control (59.6 ± 5.7 quanta) or oligomycin alone (54.9 ± 4 quanta; P_Kruskal-Wallis_ = 0.0032; Figure 6E). The increase in synaptic depression was not due to the desensitization of postsynaptic receptors, since the amplitude of mEPP did not change significantly over time in CCCP (for example, in the fiber of Figure 6, from 1.36 ± 0.27 mV to 1.34 ± 0.49 mV), or to severe failure of vesicle replenishment, since many vesicles still fused asynchronously (Supplementary Figures 1A,B).

Since ψm depolarization increases cytosolic [Ca^2+^] and [Ca^2+^] affects the priming of synaptic vesicles and the RRP size (Sakaba and Neher, 2001b; Burrone et al., 2002; Taschenberger et al., 2002; Habets and Borst, 2007; Hosoi et al., 2007; Ruiz et al., 2011; Thanawala and Regehr, 2013), we next examined the effect of CCCP on the number of available vesicles for synchronous release. For example, in the fiber of Figure 6A, the RRP decreased by ~39% over time in CCCP, from ~1,103 to ~672 quanta (Figure 6F), despite the p_r_ increase (Figure 6B, inset). On average, the size of the RRP after 5–10 min in CCCP (889.7 ± 110.3 quanta, six fibers, four mice) was significantly smaller (Figure 6G) than in control (1,962 ± 150.1 quanta, 10 fibers, eight mice) or with oligomycin alone (1,847 ± 109.2 quanta, eight fibers, six mice; P_Kruskal-Wallis_ = 0.0018).

The RRP size decrease was correlated with the concomitant increase in asynchronous release, which varied between different nerve terminals and over time in CCCP. For example, in the fiber shown in Figure 6, asynchronous release during the 1-s train accounted for ~20% of total release after 1 min in CCCP (Supplementary Figure 1C) and ~48% after 10 min (Supplementary Figure 1D). The asynchronous peak vesicle release rates went from ~614 s^−1^ to 1,145 s^−1^, and the synchronous ones from 2,429 s^−1^ to ~1,235 s^−1^.

Finally, we estimate the time constant of RRP depletion (τ_RRP_, open symbols) and the half-time of refilling (dark symbols) during synaptic activity in control, oligomycin alone, and oligomycin plus CCCP (Figure 6H). RRP loss became approximately two times faster (from ~195 to ~90 ms), and reloading three times faster (from ~370 to ~113 ms) between the first and last recording in CCCP. On the contrary, no change was observed over time in control or oligomycin alone. On average, the τ_RRP_ in control was 266 ± 19.9 ms (*n* = 10 fibers) and 276 ± 30.1 ms (eight fibers) in oligomycin alone. The average refill half-time was 433 ± 34 ms and 415.7 ± 23.5 ms, respectively. In CCCP, the velocity of the RRP loss was inversely correlated with the frequency of mEPPs before stimulation (Figure 6I, *n =* 15 terminals, six mice). The τ_RRP_ became faster within a narrow range of mEPP frequencies, reaching an estimated minimum mean value of ~129 ms for release rates ≥10 vesicles/s (Figure 6I, dotted line). Acceleration of the RRP loss in correlation to the increase in [Ca^2+^] at release sites has been previously observed in the calyx of Held (Sakaba and Neher, 2001a).

Although the above results suggested that the RRP emptying kinetics through synchronous fusions is Ca^2+^-dependent, we wondered whether the observed acceleration was due to the loss of vesicles through asynchronous fusions. Hence, we estimated the number of asynchronous events along each train and calculated the total release. Supplementary Figures 1E,F compare total quanta released (black traces), as well as that released synchronously (gray traces) and asynchronously (blue traces) during the 1-s AP train. The kinetics of RRP loss for vesicles going through the synchronous mode changed little despite the increase in asynchronous fusions. For example, the RRP depletion rate, the inverse of the time constant, for synchronous and total release after 10 min in CCCP was ~0.0111 and ~0.0092 vesicles/ms, respectively. Twice as fast as after 1 min in CCCP (~0.0053 vesicles/ms), and approximately three times faster than in control and oligomycin alone (~0.0037 vesicles/ms), indicating that the increase in asynchronous fusions along the 1-s train was not the main cause of the observed acceleration of synchronous release from the RRP.

Together, these results indicate that mitochondria limit the velocity of synchronous release under physiological conditions and participate in the regulation of synaptic plasticity during short-duration bursts of electrical activity.

## Discussion

This study examined the spatiotemporal and functional coupling between exocytosis and presynaptic mCa^2+^ at the motor nerve terminal of adult mice by a combination of techniques. Our results show that: *(i)* mitochondria and regions of high exocytosis (hot spots) are closely localized, *(ii)* mitochondria uptake Ca^2+^ upon arrival of a small number of APs at the terminal, and *(iii)* the inhibition of mCa^2+^ uptake increases the probability of release, accelerates the RRP depletion and refilling rates, and produces a rapid increase in asynchronous fusions during short bursts of APs. We propose that mitochondria regulate the synaptic response during the physiological activity of mature motor nerve terminals.

### Spatial Relationship Between Hot Spots of Exocytosis and Mitochondria

Ca^2+^ microdomains are regions of an estimated size of 100 nm^–1^ μm across (Neher and Sakaba, 2008) where [Ca^2+^] rises and decays rapidly around voltage-dependent Ca^2+^ channels (Adler et al., 1991; Stanley, 1993; Neher, 1998; Bucurenciu et al., 2008). Multiple mechanisms participate in the spatiotemporal control of Ca^2+^ in microdomains, including mitochondria, which could be considered a part of the endogenous fixed Ca^2+^ buffer system. Positioning of mitochondria relative to AZs is critical for both local Ca^2+^ buffering and immediate energy source for synaptic vesicle functionality. Reports on the location of mitochondria within CNS axon terminals and crayfish and *Drosophila* motor nerve terminals show that most mitochondria are located in a central region of synaptic boutons, containing few synaptic vesicles (Gotow et al., 1991; King et al., 1996; Chouhan et al., 2010). However, a subset of mitochondria has also been reported to be spatially closely associated with clusters of synaptic vesicles at mouse motor nerve terminals (Torres-Benito et al., 2011, 2012) and the calyx of Held (Wimmer et al., 2006), forming rings or donut-like structures. Electron tomography in the calyx of Held synapse also shows mitochondria connected to the presynaptic membrane near AZs through cytoskeletal structures (Perkins et al., 2010). Our live imaging experiments showed spatial association of hot spots of exocytosis and mCa^2+^ signals (Figure 2); however, the relatively low resolution of our imaging system precludes us from estimating more accurately the distance between these two signals. Nevertheless, we started to detect an increase in mCa^2+^ with ~5–10 AP (Figure 3D), that is, ~50–100 ms from the onset of a train at 100 Hz (Figure 3C), in agreement with the findings that neuronal mitochondria can uptake Ca^2+^ in the submicromolar range (Chouhan et al., 2010; Ashrafi et al., 2019) and that a single AP can trigger mCa^2+^ influx at hippocampal neurons boutons (Gazit et al., 2016). In synapses, the high sensitivity of mitochondria to Ca^2+^ may be relevant not only to preventing Ca^2+^ accumulation in microdomains and their surroundings but also to modulate the range of responses to physiological stimuli during development and maturity. For example, in the mouse calyx of Held, mitochondrial volumes are increased to support high firing and secretion rates upon maturity (Kim et al., 2013; Thomas et al., 2019) and in *Drosophila* motor nerve terminals, presynaptic mitochondrial volume and packing density scale with presynaptic demands (Justs et al., 2022).

### The Role of Mitochondria on the RRP Size and the Release Kinetics

We previously observed that the size of the functional (effective) RRP is frequency-dependent; the higher the stimulation frequency, the larger the RRP (Ruiz et al., 2011). An interpretation of this result is that the size of the RRP partially depends on the accumulation of Ca^2+^ during repetitive stimulation (Zucker and Regehr, 2002; Neher and Sakaba, 2008; Thanawala and Regehr, 2013). Under this hypothesis, one might expect that mitochondrial depolarization will produce a larger accumulation of Ca^2+^ in nerve terminals and, concomitantly, an increase in the effective RRP size. However, we did not observe an enhancement of the RRP but a decrease (Figures 6F,G). On one side, the no increase in the RRP agrees with our previous observation that the maximum size of the RRP at this synapse comprises ~1,700–2,000 vesicles, with no further increase when the stimulation frequency is above 50 Hz (Ruiz et al., 2011). Therefore, our present results also indicate that the upper limit of the RRP size in this synapse is reached at 50 Hz stimulation and that the reduction in mCa^2+^ uptake cannot increase it. On the other side, the progressive decrease in the RRP with CCCP could be due, among others, to the increase in asynchronous fusions (Figure 5E, Supplementary Figures 1C,D), reduction in the mitochondrial ATP production (Justs et al., 2022), partial inactivation of P/Q-type voltage-dependent Ca^2+^ channels by high Ca^2+^ (Forsythe et al., 1998; Demaria et al., 2001), moderate desensitization of postsynaptic receptors, or a combination of all. In all cases, mCa^2+^ uptake could minimize these effects during physiological stimuli.

Interestingly, together with the decrease in the RRP, we observed faster vesicle depletion and recruitment rates when the mCa^2+^ uptake was inhibiting (Figure 6H), suggesting a role of mitochondria in the regulation of these two processes. These observations agree with previous findings showing Ca^2+^-dependent acceleration of vesicle recruitment in numerous types of synapses, including the calyx of Held (Wang and Kaczmarek, 1998; Sakaba and Neher, 2001a), the climbing fiber to cerebellar Purkinje cell synapses (Dittman and Regehr, 1998), excitatory hippocampal synapses (Stevens and Wesseling, 1998), the ribbon synapse in the retina (Von Gersdorff et al., 1998; Gomis et al., 1999), and the NMJ (Ruiz et al., 2011). Besides Ca^2+^, the rate of vesicle recruitment at the steady-state is shortened by raising the temperature from 23°C to 37°C, both at the calyx of Held (Kushmerick et al., 2006) and the NMJ (Ruiz et al., 2011), what has no appreciable effect on the initial RRP size. These findings indicate that Ca^2+^ and temperature are two major determinants of short-term depression during high-frequency firing.

### Mode of Fusion Change Upon mCa^2+^ Uptake Inhibition

The inverse synchronous and asynchronous release occurrence observed in our experiments during short AP trains (1-s) when the mCa^2+^ uptake was inhibited with CCCP (Figures 5B,C, and Supplementary Figure 1) suggests a switch in the mode of fusion of vesicles belonging to the RRP (Hagler and Goda, 2001; Otsu et al., 2004). Alternatively, or additionally, asynchronous fusions could result from secondary docked vesicles (Nagwaney et al., 2009) located outside AZ (Schneggenburger and Neher, 2005; Wen et al., 2013) and newly recruited vesicles from other pools that compete for the same release sites.

It has been found that synaptic vesicles are functionally and molecularly heterogeneous within the presynaptic terminal (Sakaba and Neher, 2001b; Hua et al., 2011) and that they could be spatially segregated according to their molecular identities (Wen et al., 2010). For example, asynchronous release has been reported to be triggered by a relatively low [Ca^2+^] (Otsu et al., 2004), indicating that vesicles that use this mode of release possess a high-affinity Ca^2+^ sensor (Sugita et al., 2002). Supposing that the vesicles that fuse asynchronously are located at a greater distance from voltage-dependent Ca^2+^ channels than the synchronous pool, it is expected they fuse after a relatively prolonged elevation of Ca^2+^ within microdomains. Alternatively, Ca^2+^ accumulation at microdomains during sustained activity may promote the integration of Synaptotagmin 7 and other synaptic proteins related to asynchronous release (Virmani et al., 2003; Sun et al., 2007) into the vesicular membrane *via* endocytosis, changing release towards the asynchronous mode while using the same release sites. In any case, controlling Ca^2+^ accumulation at the microdomains and their surroundings is crucial for determining the release mode.

In summary, our results suggest that mitochondria and release sites are organized in functional subsynaptic compartments for the spatial and temporal regulation of release during physiological neuronal activity. We propose that mitochondria uptake Ca^2+^ with high sensitivity and play a significant role in maintaining synaptic transmission strength and reliability in the NMJ under physiological activity.

## Data Availability Statement

The original contributions presented in the study are included in the article/Supplementary Material, further inquiries can be directed to the corresponding author.

## Ethics Statement

The animal study was reviewed and approved by Ethics committee of the University of Seville and Junta de Andalucia.

## Author Contributions

ML-M, AF-M, and LT designed experiments, edited and approved the manuscript. ML-M and LT performed and analyzed live fluorescence imaging experiments. AF-M and LT performed and analyzed electrophysiological experiments. All authors contributed to the article and approved the submitted version.

## Conflict of Interest

The authors declare that the research was conducted in the absence of any commercial or financial relationships that could be construed as a potential conflict of interest.

## Publisher’s Note

All claims expressed in this article are solely those of the authors and do not necessarily represent those of their affiliated organizations, or those of the publisher, the editors and the reviewers. Any product that may be evaluated in this article, or claim that may be made by its manufacturer, is not guaranteed or endorsed by the publisher.

## Footnotes

^1^ https://idus.us.es/bitstream/handle/11441/24203/S_TD_PROV162.pdf?sequence%20=%201&isAllowed%20=%20y

## Supplementary Material

Supplementary Figure 1 Change of the release mode over time in the presence of ψm depolarization. **(A,B)** Cumulative release components, asynchronous **(A)** and synchronous **(B)**, during 1-s train stimulation (50 Hz) after 1 min (blue lines) and 10 min (red lines) in CCCP (2 μM). **(C,D)** Comparison of cumulative total release (black) and synchronous release (gray) after 1 min **(C)** and 10 min **(D)** in CCCP calculated from **(A,B)**. **(E,F)** Time course of total (black), asynchronous (gray) and asynchronous (blue) quanta released during 1-s AP train.
