# Supplementary figures and images for: Presynaptic Mitochondria Communicate With Release Sites for Spatio-Temporal Regulation of Exocytosis at the Motor Nerve Terminal
Source: Front Synaptic Neurosci. 2022 May 12;14:858340. doi: 10.3389/fnsyn.2022.858340 (PMC9133601; doi:10.3389/fnsyn.2022.858340)

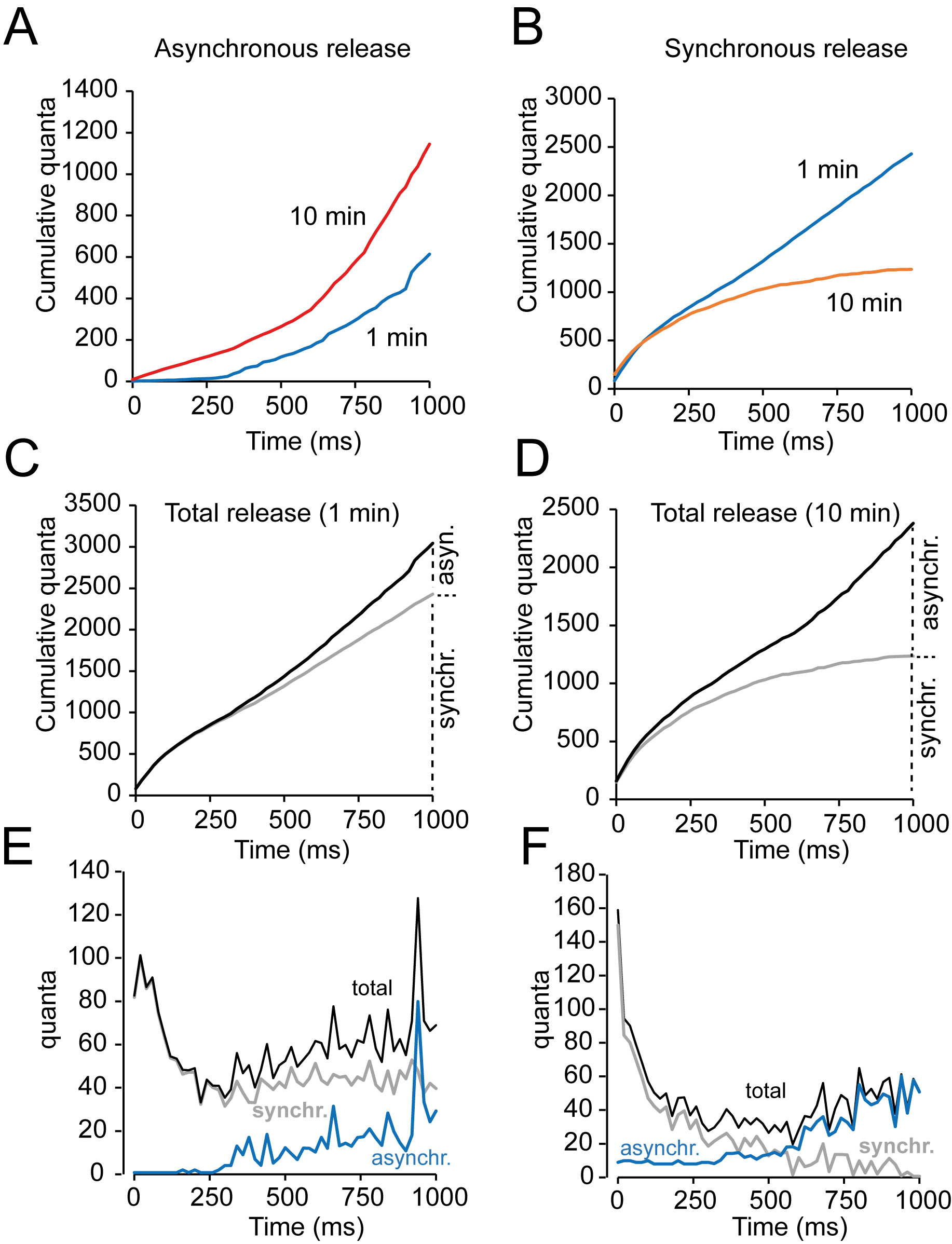

Supplement: Supplementary Figure 1 — Change of the release mode over time in the presence of ψm depolarization. (A,B) Cumulative release components, asynchronous (A) and synchronous (B), during 1-s train stimulation (50 Hz) after 1 min (blue lines) and 10 min (red lines) in CCCP (2 μM). (C,D) Comparison of cumulative total release (black) and synchronous release (gray) after 1 min (C) and 10 min (D) in CCCP calculated from (A,B). (E,F) Time course of total (black), asynchronous (gray) and asynchronous (blue) quanta released during 1-s AP train. [file Image_1.TIF]
